# Considering Situational Variety in Contextualized Aging Research – Opinion About Methodological Perspectives

**DOI:** 10.3389/fpsyg.2021.570900

**Published:** 2021-04-12

**Authors:** Friedrich Wolf, Alexander Seifert, Mike Martin, Frank Oswald

**Affiliations:** ^1^Interdisciplinary Ageing Research (IAW), Department of Educational Sciences, Goethe University Frankfurt, Frankfurt, Germany; ^2^Center for Gerontology, University of Zurich, Zurich, Switzerland; ^3^School of Social Work, University of Applied Sciences and Arts Northwestern Switzerland, Olten, Switzerland; ^4^University Research Priority Program “Dynamics of Healthy Aging”, University of Zurich, Zurich, Switzerland

**Keywords:** context, aging, situation, ambulatory assessment, real-life study, experience sampling

## Abstract

Due to the increasingly heterogeneous trajectories of aging, gerontology requires theoretical models and empirical methods that can meaningfully, reliably, and precisely describe, explain, and predict causes and effects within the aging process, considering particular contexts and situations. Human behavior occurs in contexts; nevertheless, situational changes are often neglected in context-based behavior research. This article follows the tradition of environmental gerontology research based on Lawton’s Person-Environment-Interaction model (P-E model) and the theoretical developments of recent years. The authors discuss that, despite an explicit time component, current P-E models could be strengthened by focusing on detecting P-E interactions in various everyday situations. Enhancing Lawton’s original formula via a situationally based component not only changes the theoretical perspectives on the interplay between person and environment but also demands new data collection approaches in empirical environmental research. Those approaches are discussed through the example of collecting mobile data with smartphones. Future research should include the situational dimension to investigate the complex nature of person environment interactions.

## Introduction

Trajectories of aging are inter- and intra-individually, but also inter- and intra-contextually heterogeneous, complex, and diverse. To understand and explain the elements and processes that guide the development of this heterogeneity, Gerontology requires theoretical models and empirical methods that can meaningfully, reliably, and precisely describe, explain, and predict causes and effects within the complexity of the aging process and the factors influencing it within and across contexts and situations. Such models provide a theoretically informed basis to understand and explain aging in the real world. These, in turn, can be used for individualized, contextualized, and situation-aware interventions and support services. As human behavior happens in social and spatial contexts, it cannot be explained solely by personal factors, such as personality and competence, but must also be explained by contextual factors. In addition, as individuals do not (re)act the same way over time even in similar situations, the interactions between person, context, and situation are crucial to the understanding of the heterogeneity of aging. Therefore, this research will argue that there is potential for further development of a “situation- and context-considering” view in aging research.

## Origins of Environmental Gerontology: Lewin and Lawton

Gerontology has a long tradition of addressing contexts in behavioral research. For instance, “environmental gerontology” is influenced by the psychological concepts of person-environment (P-E) exchange. M. Powell Lawton and Lucille Nahemow (1973) stated that the environment has a non-negligible influence on the aging process and individual behavior. This rationale reaches back to the field theory of Kurt Lewin (1951), which can be expressed as a mathematical formula:

B=f⁢(P,E)

This formula means that the behavior of a person (B) is a function of that person’s traits and the environment in which he or she operates (Lewin, 1951; Beckmann and Heckhausen, 2018, p. 122). For Lewin, individual behavior was based on a person’s competence and cognitively represented environmental features; here, he has already addressed the need to emphasize the situation with his term “field at a given time” (Lewin, 1951, p. 42). Thus, people of similar competence, exposed to the same environment, will behave similarly.

Lawton took Lewin’s formula and refined it to predict older adults’ behavior (Lawton and Nahemow, 1973; Lawton, 1982). In his environmental press–competence model, Lawton added an interaction term (PxE) to the formula:

B=f⁢(P,E,P×E)

This term means that behavior is also based on an interaction between a person (P) and the environment (E). Lawton, thus, expanded Lewin’s formula to include observations showing that similar competence under the same environmental press (or richness) does not necessarily generate similar behavior. This also implies that individuals with identical abilities and identical environments can generate a large variety of (docile or proactive) behaviors, the amount of variety largely depending on the number of opportunities in a given environment. From the observed range of real-world behaviors, it is, in most instances, impossible to determine the underlying competence. So, in fact, PxE is an additional source of information that cannot be predicted solely based on the person (P) and the environment (E) characteristics but indicating sensitivity for a range of possible interactions (x). PxE expresses individual styles, individuals’ cognitive representations of the environment, and the accompanying effects influencing behavior (Lawton, 1982). In contrast to Lewin, Lawton emphasized broadly defining the environment, including the non-physical environment, extra-individual stimuli, and intraindividual representations of the environment in the PxE formula to reliably predict older adults’ behavior. In summary, Lewin considered the situation as a relevant factor but did not make it explicit, while Lawton focused more on the variability of P and E and implied that similar P-E constellations also lead to similar behavior. However, Lawton himself adapted the model consequently with respect to specify and differentiate processes of docility versus proactivity or to include meanwhile existing empirical evidence, e.g., in the field of housing (Lawton, 1989, 1998), but did not implement a situational component.

## Current Pxe Models in Gerontological Research

Recently, Wahl et al. (2012) have taken Lawton’s theory and developed a new integrative framework, including objective and subjective person–environment interactions. They focus primarily on specifying PxE processes and aging-related developmental outcomes, such as identity, autonomy, and wellbeing (Wahl and Oswald, 2016; Oswald and Wahl, 2019). The context is defined as socio-spatial, including physical, social, and digital environments. The latter opens a new “space” and is quickly increasing in significance for the aging process. The core of this concept is that PxE interactions can be classified into two central processes: belonging and agency, building on the “Social-Physical-Places-Over-Time” model (SPOT, Wahl and Lang, 2004). PxE agency includes all goal-directed behaviors related to the physical, social, and digital environments. The individual both reacts to and proactively acts in the environment. Belonging refers to subjective evaluations and interpretations of the environment. The authors extend Lawton’s model via the central factor of belonging, which must be empirically considered to predict behavior and central gerontological outcomes, such as autonomy, identity, and wellbeing (Oswald and Wahl, 2019).

Wahl and Gerstorf (2018) advance this with their Context Dynamics in Aging (CODA) framework. The central innovation they make to previous PxE models is the explicit division of proximal and distal contexts. Proximal contexts are those with which older individuals directly interact – ranging from concrete technical devices to regularly used benches on the way to the supermarket. Distal contexts are those with which older individuals indirectly interact – for example, digital social infrastructures, which automatically change communication behaviors in broad population groups and, in turn, affect individuals’ lives. Though Wahl and Gerstorf (2018) do focus on the aging individual, perspectives on the situational PxE interactions processes remain unexplored. This is unfortunate because it would be very relevant to closely examine the situational significance of proximal and distal environments and better understand the relevance of particular contexts to situational behavior.

Chaudhury and Oswald (2019) have expanded Wahl and Oswald’s (2016) PxE exchange model with respect to (1) exploring the primary components and interactions of individual and environmental features, (2) digging deeper into the nature of agency and belonging with respect to time, and (3) considering autonomy and identity as intermediate outcomes in more detail. In sum, they provide a situational framework that is easy to use for empirical environmental gerontology research. The individual and environmental features of these extensions have been divided into individual characteristics (e.g., physical health or cognitive status), social factors (e.g., social support or living arrangements), components of the physical and built environment (e.g., homes or neighborhoods) and technological systems (e.g., ambient and wearable sensors or handheld devices). Chaudhury and Oswald (2019) also argue that the dialectical processes of agency and belonging can be represented in various dimensions, such as independent functioning, social interaction/connection, privacy, mobility, safety and security, or continuity of the self. Moreover, they emphasize a specific time-based component to PxE interactions, not only referring to lifespan, but also addressing the small-scale timeframes of everyday P-E exchange – such as hours, days, weeks, or months – and assuming a dynamic interaction and tension between the two psychological forces at any given time.

In summary, current models have extended Lawton’s P-E interaction term significantly in the recent years. They have included the complexity of the aging process by considering variations and interaction terms of P and E. However, these models can be strengthened by adding a situational component. Consequently, we suggest adding a situation term, “s,” to Lawton’s formula, with the consequence that the complex P-E interaction processes, described in the current models, also need to be situationally assessed. The following paragraphs suggest how adding a situational factor to Lawton’s formula affects current theory in environmental gerontology and extends empirical and statistical approaches for assessing everyday P-E interactions.

## Lawton Revisited

Connecting existing PxE models with the real-life approach of experience sampling (Brose and Ebner-Priemer, 2015) leads us to conclude that Lawton’s formula remains a sufficient guideline for gerontological research addressing context but that it should also be complemented by a situational factor:

$$B_{i,s}=f\,\left(P_{i,s},E_{i,s};P_{i,s,\times }\,E_{i,s}\right)$$

This change meets the requirement that the reciprocal relation between context (E) and person (P) should differ across situations (s) and should, therefore, be measured repeatedly and continuedly to address different situational PxE interactions.

The PxE exchange could be understood as a two-level model. Level one (s) addresses behavioral variations between situations, and level two (i) addresses behavioral variations between persons. These changes also account for the fact that older adults may react dynamically to different situations, breaking the methodological stereotype that they always react similarly in similar contexts. Therefore, the formula and the multi-level models based on it increasingly consider the lived reality of older people and real-life situational chances. This approach supports the idea of *individualizing* later-life interventions – i.e., adapting them to the abilities or traits of a person – and, at the same time, *contextualizing* later-life interventions – i.e., adapting them to situational requirements.

The importance of this distinction becomes obvious when applied to situation-aware assistive systems (e.g., Krüger et al., 2014; Yordanova et al., 2017). Translating the authors’ suggested theoretical extension to include situational information by applying machine-readable data interpretation systems (ontologies), these groups have succeeded, for instance, in detecting, with great precision, situational instances of real-life disorientation in older persons. Schaat et al. (2020) have shown that individuals with a diagnosis of dementia, when observed in real life, behave in a disoriented way often in less than 10% of situations. Without the extension proposed here, an individualized intervention for persons with dementia would have suggested an orientation intervention for all individuals in this group based on the group’s significantly higher level of disorientation compared to individuals without dementia. However, this intervention would be situationally inadequate for each member of this group in 90% of all situations. Thus, with the help of this theoretical extension, the authors have provided the basis for systematically developing individualized and contextualized interventions, which can much more specifically target intervention situations in which large effects are likely.

Although the current PxE models repeatedly point out the significance of considering time in PxE processes, the basic assumption is still that these exchanges are rather stable and that they change primarily through major shifts in P or E. This raises the question of how much situational variance can be found in domain-specific PxE processes. The hypotheses to be pursued here are that the domain influences intraindividual variance, that individual traits influence intraindividual variance, and that the interaction between the domain and individual traits influences intraindividual variance in situational behavior.

## Further Research: Consider the Situation

As already shown by the extension of Lawton’s formula, considering the situation in future gerontological research does not simply imply including linear time as an explanatory variable. It should also be emphasized that situation is not equivalent to context. Situation is the individual constellation of personal characteristics and environmental factors at a specific point in time. To explain the behavior of a person in its full complexity, an attempt must, therefore, be made to measure the constellation of variables that form a specific situation and use statistical methods to relate them mathematically to the behavior shown.

The hypothesis behind these considerations is that each element of the person–environment interaction can vary, and each variation leads to different interactions between P and E, resulting in potentially infinite PxE constellations. New insights are possible by asking which constellations are associated with which behaviors. The assumption that each PxE constellation leads to a specific behavior should also be questioned. There are many situations in which different PxE constellations lead to the same behavior. By breaking down the PxE variations in detail, significant factors for the actual behavior can be identified under the control of all other PxE variables.

Instead of referring to (to our knowledge not yet) existing empirical evidence an example may illustrate the importance of situational measurement in PxE interactions. Imagine an older woman, who visits a park in her neighborhood every day – enjoying nature, peace, and quiet for about half an hour. The outcome is her affective wellbeing, caused by enjoying the park. Though the PxE constellations are always the same, significant variance is seen in affective wellbeing. Therefore, either the two events, wellbeing and the park visit, are independent, or the PxE parameter variations cause variations in affective wellbeing. This question can be answered by measuring the PxE parameters every day. The situation can be influenced by external environmental factors (e.g., noise or weather), factors preceding the PxE constellation (e.g., an argument between the woman and her partner before she left home), or factors within the person (e.g., pain or discomfort). According to our proposed revision of Lawton’s formula, in every situation, the individual factors must be considered separately, but their interactions must also be considered. Some factors may have stronger combined effects than others. The negative feelings brought on by an argument can be relativized in the park. Pressure in the chest may be perceived more intensely. This answers whether visiting the park influences affective wellbeing and also shows in which situations the park can have a positive influence and in which situations this positive influence may be overridden.

Our own empirical research in the fields of social interactions and experience of neighborhoods as well as smartphone use in everyday life already follow this approach and are designed to reflect the particular situation as accurately as possible (Wolf, 2018; Seifert, 2020). Measuring PxE constellations and interactions in situations has become increasingly practicable in recent years, especially with the spread of smartphones (e.g., Wolf, 2018; Demiray et al., 2019; Luo et al., 2019; Seifert, 2020). Smartphone mobile data collection enables innovative gerontological research considering context and situation (Martin et al., 2018; Seifert and Harari, 2019). Particularly when analyzing contextual and situational factors, it seems increasingly important to capture intraindividual representations of the physical, social, and digital environments. This can generate a better understanding of situational belonging processes and provide an empirical basis for current PxE models, valuably contributing to the further development of existing theories. Future gerontological research should make greater use of methods capturing situation-specific PxE interactions. For example, Seifert et al. (2018) describe the potential and pitfalls of mobile data collection via smartphones, which presents two challenges for future gerontological research: the methodological challenge of designing research with multi-level models and the challenge of specifying theoretical situation-models focusing strongly on real world PxE interaction situations. The former leads to the conclusion that ambulatory assessments should be increasingly used in data collection and that, at best, multi-level models for environmental gerontological research should become the standard for statistical methods. The latter takes a step forward. Since many theoretical models assume there are potentially different outcomes for different P and E combinations, more attention should be given to the extent to which identical P and E combinations result in different behavioral outcomes. This opens a completely new field of research that, instead of examining context-independent predictors of behavioral limitations, describes and explains the intrapersonal and inter-situational heterogeneity of behaviors as a potential predictor of healthy aging. This is a challenge for theory building as well as for future assessment procedures of empirical data, but it offers the opportunity to improve reflection on and evaluation of statistical effects.

Beside the more quantitative approaches described above, it is also important to use this situational variety in contextualized aging research for qualitative research approaches, e.g., qualitative interviews (Windisch et al., 2018). In addition to interview methods, ethnographic research approaches should also be mentioned at this point. Participatory observation in particular focuses on the description of complex PxE interactions (e.g., Clark et al., 2009). The complexity of PxE processes also offers interesting opportunities for mixed-methods methodology, e.g., by using the patterns in quantitative data to explain and contextualize them through interviews.

## Conclusion

Lawton’s theory – that human behavior can only be adequately explained if characteristics of both P and E are considered – is still valid for environmental psychology and also for gerontological research. Recent environmental gerontological theories have further developed Lawton’s formula, particularly regarding PxE. The nature and form of interaction processes have been theoretically specified, and contexts important to the aging process have been identified. Nevertheless, the models still lack a clearly defined, situation-related component. Therefore, this research argues for an essential expansion of existing PxE models by adding the term *s* to represent behavioral variations between different situations in the same contexts. It asserts that empirical environmental gerontology should include this term in considerations of older adults’ contextually influenced behavior. It also recommends a greater use of mobile data collection via smartphones, which are extremely versatile survey instruments, to map situational and contextual influences on behavior, autonomy, and wellbeing in a more detailed way. Research would profit from increasingly complementing person-centered models of aging with situational models of aging to explain the variability of behaviors across concrete, everyday situations and emphasize statistical explanations of intraindividual variance in aging-related outcomes.

## Author Contributions

FW conceptualized the article and wrote the initial draft. All authors have jointly taken up the concept and developed it further.

## Conflict of Interest

The authors declare that the research was conducted in the absence of any commercial or financial relationships that could be construed as a potential conflict of interest.
